# Effective Prediction of Mortality by Heart Disease Among Women in Jordan Using the Chi-Squared Automatic Interaction Detection Model: Retrospective Validation Study

**DOI:** 10.2196/48795

**Published:** 2023-07-20

**Authors:** Salam Bani Hani, Muayyad Ahmad

**Affiliations:** 1 Clinical Nursing Department School of Nursing The University of Jordan Amman Jordan

**Keywords:** coronary heart disease, mortality, artificial intelligence, machine learning, algorithms, algorithm, women, death, predict, prediction, predictive, heart, cardiology, coronary, CHD, cardiovascular disease, CVD, cardiovascular

## Abstract

**Background:**

Many current studies have claimed that the actual risk of heart disease among women is equal to that in men. Using a large machine learning algorithm (MLA) data set to predict mortality in women, data mining techniques have been used to identify significant aspects of variables that help in identifying the primary causes of mortality within this target category of the population.

**Objective:**

This study aims to predict mortality caused by heart disease among women, using an artificial intelligence technique–based MLA.

**Methods:**

A retrospective design was used to retrieve big data from the electronic health records of 2028 women with heart disease. Data were collected for Jordanian women who were admitted to public health hospitals from 2015 to the end of 2021. We checked the extracted data for noise, consistency issues, and missing values. After categorizing, organizing, and cleaning the extracted data, the redundant data were eliminated.

**Results:**

Out of 9 artificial intelligence models, the Chi-squared Automatic Interaction Detection model had the highest accuracy (93.25%) and area under the curve (0.825) among the build models. The participants were 62.6 (SD 15.4) years old on average. Angina pectoris was the most frequent diagnosis in the women's extracted files (n=1,264,000, 62.3%), followed by congestive heart failure (n=764,000, 37.7%). Age, systolic blood pressure readings with a cutoff value of >187 mm Hg, medical diagnosis (women diagnosed with congestive heart failure were at a higher risk of death [n=31, 16.58%]), pulse pressure with a cutoff value of 98 mm Hg, and oxygen saturation (measured using pulse oximetry) with a cutoff value of 93% were the main predictors for death among women.

**Conclusions:**

To predict the outcomes in this study, we used big data that were extracted from the clinical variables from the electronic health records. The Chi-squared Automatic Interaction Detection model—an MLA—confirmed the precise identification of the key predictors of cardiovascular mortality among women and can be used as a practical tool for clinical prediction.

## Introduction

### Background

Cardiac disease covers a range of cardiac conditions, including heart attacks and coronary artery disease [1]. Heart disease is sometimes considered a male illness, but the fact that women die of heart disease at the same rate as men each year contradicts this notion [2]. According to the Centers for Disease Control and Prevention, about 56% of women recognize that heart disease is their leading cause of death [3]. Heart disease was reported to be the primary cause of death among women in the United States in 2020 [4]. For more precise results, it has been found that the most prevalent form of heart disease, coronary heart disease, affects approximately 1 in 16 (6.2%) women aged 20 years and older [5]. Recent data indicate a stall in the declines in coronary heart disease incidence and mortality, especially in younger women aged <55 years [6]. Furthermore, new issues had emerged in transitional countries as a result of globalization, which increased risk factors and sedentary lifestyle adoption, having sharply increased cardiovascular mortality rates [7].

Sex chromosomes alter gene expression, which may then be further altered by sex-specific hormonal variations, resulting in sex-specific cardiovascular gene expression and function [6]. These differences result in variations in the prevalence and manifestation of cardiovascular disorders, including those related to autonomic regulation, hypertension, diabetes, and vascular and cardiac remodeling [8].

Age, smoking, obesity, high blood pressure, pulse, mean arterial pressure, diabetes, cholesterol, poor diet, and lack of physical activity are the primary risk factors for heart disease [9]. Many clinical examinations are available to diagnose coronary heart disease, including electrocardiography, cardiac enzyme assays, x-ray imaging, and angiography [10]. The data stored in the electronic health system of a health care organization generates a vast amount of unanalyzed raw data that aid in the prevention and treatment of cardiovascular disease [11].

Currently, machine learning algorithms (MLAs), as a specific artificial intelligence modality, are playing an important role in the field of disease prediction including cardiology mortality prediction, big data storage, acquisition, and recovery as primary prevention strategies [12,13]. A widespread MLA data set is used to predict mortality among women—through what is known as data mining—to determine significant features of variables that assist in detecting the main causes of mortality in this target group of the population since such risk prediction is an integral aspect according to the international guidelines of primary prevention of heart disease [14,15]. Thus, understanding the unique aspects of predicting mortality due to heart disease in women, including a lower incidence of heart disease due to a later age of heart disease occurrence, development of new prediction models, and differences in the effects of laboratory data—known as biomarkers—are critical factors in predicting mortality due to heart disease. However, there is a scarcity of studies that effectively predict mortality caused by heart disease among women. Hence, this study aims to effectively predict heart disease among women, using the MLA data set.

### Research Questions

Our research questions are as follows: (1) what are the risk factors for the development of heart disease in Jordanian women, that are related to death versus a life status? (2) What is the best model to use to generate the best performance metrics based on the data extracted from electronic health records (EHRs)?

## Methods

### Study Design

The study used a retrospective design in retrieving data from Electronic Health Solutions (EHS) for Jordanian women with heart disease. Data were collected for Jordanian women who were admitted to public hospitals from 2015 to 2021.

### Study Variables

Patients’ age, geographic location (governorate), medical diagnosis based on the International Classification of Disease, Tenth Revision (ICD-10), laboratory findings including high-density lipoprotein (HDL), lactate dehydrogenase (LDH), cholesterol level, fasting blood sugar (FBS), systolic blood pressure (SBP), and diastolic blood pressure (DBP) were obtained from the health data analytics department for the admitted patients for the period of 2015 to the end of 2021. Data were downloaded as Excel (Microsoft Corp) sheets in many files for health data analysis. None of the data on age, medical diagnosis, and place of residence were missing. However, there were numerous missing values for variables of LDH, HDL, cholesterol, glycated hemoglobin (HbA_1c_), creatinine, FBS, and vital signs including oxygen saturation (measured with pulse oximetry), heart rate, SBP, and DBP. Mean arterial pressure (MAP) and pulse pressure were calculated. Data were merged in a single file using the SPSS program [16] and then sorted and cleaned.

### Data Source

After receiving approval to extract the necessary data, which took about 6 months, the health data analytics department that stores the EHRs was contacted. Unfortunately, we encountered some extraction challenges that made it difficult to gather all necessary data. Electrocardiography and catheterization reports are 2 examples of data that could not be obtained because they require a natural language processing method that is not available in the EHR system. In terms of physical information, the system lacked the patients' height, weight, and ejection fraction (percent), as well as information on occupation and dietary habits.

### Data Analysis

#### Data Processing

Using frequency analysis and outlier detection, the data were examined for noise, inconsistency, and missing values. A large number of redundant data were removed after sorting, cleaning, and organizing the extracted data.

#### Data Transformation

The researchers chose the most relevant attributes of coronary heart disease using data visualization. Moreover, SPSS Modeler (version 18.0; IBM Corp) [16] was used for manipulating, analyzing, and visualizing the data, which provides the features of presenting the data with high statistical power and predictive analysis and data management for descriptive and predictive modeling [17]. Descriptive modeling was used to identify the main risk factors for heart disease that lead to death. Furthermore, predictive modeling was used to build the appropriate model based on the overall accuracy and area under the curve (AUC).

#### Building Appropriate Model

This study applied the SPSS Modeler software application (IBM Corp) that helps users build and deploy predictive models. Data were imported and prepared after processing and transformation. Thereafter, SPSS Modeler offers a wide range of modeling techniques and algorithms to choose from, including decision trees, neural networks, regression, clustering, association rules, and more. The appropriate model was selected on the basis of data quality, problem type, and goals of the study. Once the model (Chi-squared Automatic Interaction Detection [CHAID]) was selected, the SPSS Modeler interface was configured to build the model. This involved specifying the input variables, target variables, and model parameters. Performance metrics including accuracy and AUC were used to evaluate the selected model.

However, the model in this study could be improved if the missing data were handled properly using proper imputation techniques. Overall, SPSS Modeler provides a comprehensive platform for data preparation, model building, evaluation, and deployment, making it a popular choice for data mining and predictive analytics tasks.

### Ethical Considerations

The Committees of Scientific Research and Ethics of Research at the School of Nursing, The University of Jordan, as well as the ethics committee at the Ministry of Health (#MOH/REC/2022/3) provided their approval for the study to be carried out in a manner that complies with ethical standards. In addition, the Health Data Analytical Department at EHS provided their approval to the study. Patients’ records were handled with confidentiality and anonymously using an ID as the distinguishing characteristic of each record. The data that were extracted were stored in a separate file that was locked up and stored in a secure location within the researchers' office.

## Results

### Sample Characteristics

The participants' average age was 62.6 (SD 15.4) years. In the extracted file of the women, angina pectoris was the most common diagnosis (n=1264, 62.3%), followed by congestive heart failure (n=764, 37.7%). The majority of the women lived in Amman, the capital of Jordan (n=1308, 64.5%), followed by Zarqa (n=257, 12.7%) and Irbid (n=153, 7.5%), which constituted the country's northern areas. A smaller percentage of women lived in Karak (n=66, 3.3%), Maam (n=24, 1.2%), and Aqaba (n=21, 1.0%), which constitute the southern regions of the kingdom (Table 1).

The laboratory findings, including LDH, HDL, cholesterol, HbA_1c_, creatinine, and FBS, were recorded. Vital signs, including oxygen saturation (measured with pulse oximetry), heart rate, SBP, DBP, pulse pressure, and MAP were used to stratify the patients’ outcomes. Unfortunately, not all patient health records had all the workup results in their EHR. Table 2 displays the minimum, maximum, mean, and SD values of the clinical and laboratory results.

**Table 1:** Sample characteristics (N=2028).

Characteristics			Values
Age (years), mean (SD)			62.6 (15.4)
**Medical diagnosis, n (%)**			
	Angina pectoris	1264 (62.3)	
	Congestive heart failure	764 (37.7)	
**Governorate, n (%)**			
	Irbid	153 (7.5)	
	Ajloun	2 (0.1)	
	Jarash	24 (1.2)	
	Mafraq	20 (1.0)	
	Balqa	104 (5.1)	
	Amman	1308 (64.5)	
	Zarqa	257 (12.7)	
	Ma’daba	48 (2.4)	
	Karak	66 (3.3)	
	Tafilah	1 (0.01)	
	Maan	24 (1.2)	

**Table 2:** Work-up results and vital signs of the patients.

Findings	Participants, n	Minimum value	Maximum value	Values, mean (SD)
Lactate dehydrogenase (IU\L)	57	124.0	816.0	246 (141.5)
High-density lipoprotein (mg/dL)	41	11.9	69.30	26.8 (14.2)
Cholesterol (mg/dL)	54	46.4	319.0	127 (64.4)
Glycated hemoglobin (%)	191	2.15	22.30	3.98 (2.55)
Creatinine (mg/dL)	228	0.02	122.0	2.29 (10.24)
Fasting blood sugar (mmol/L)	271	18.0	977.1	136.9 (110.7)
Oxygen saturation (%)	421	36	100.0	93.9 (6.59)
Heart rate (beats per minute)	1440	19	170.0	81.4 (14.4)
Systolic blood pressure (mm Hg)	2028	72	250.0	149.5 (26.5)
Diastolic blood pressure (mm Hg)	2028	41	165.0	82.6 (15.7)
Pulse pressure (mm Hg)	2028	17	137.0	66.9 (21.6)
Mean arterial pressure (mm Hg)	2028	60.6	183.0	104.7 (17.2)

### Predictive Model

The CHAID model demonstrates the most accurate model, out of 9 models, to predict death versus life status among women with heart disease, with an overall accuracy of 93.25% and AUC of 82.5% (Table 3).

The CHAID model helps in analyzing the given data to understand the main characteristics that are mostly associated with a given outcome or being a member of a target group. The results of the CHAID model are presented skillfully for interpretation as a decision tree graph [18]. The CHAID model merges the values of the target variable that is deemed to be statistically homogeneous while retaining all heterogeneous values. The first branch of the decision tree is then constructed using the best predictor, with each child node containing a set of uniform values from the selected field. The statistical test that is used depends on the target field's level of measurement, and this process is repeated until the tree is fully developed [19]. In addition, this model is similar to the other MLA as it divides the sample data into 70% (testing data set) and 30% (training data set) to determine whether the model generates reliable results.

**Table 3:** Seven models built for the study data.

Model	Overall accuracy (%)	Area under the curve
CHAID^a^	93.25	0.825
C5	93.09	0.500
Quest	93.09	0.500
C&R^b^ tree	93.09	0.500
Discriminant	75.35	0.733
Decision list	46.79	0.699
Bayesian network	17.85	0.657
Neural network	17.26	0.534
Logistic regression	16.96	0.481

^a^CHAID: Chi-squared Automatic Interaction Detection.

^b^C&R: Classification and Regression.

The 17-node model was created using the SPSS Modeler. Multimedia Appendix 1 shows that the graph of the study, as produced by the interactive CHAID tree's beginning node, branched to 4 nodes (nodes 1-4) based on tissue oxygenation (measured with pulse oximetry) with a cutoff value of 93% since women who had an oxygen saturation of ≤93% were at a high risk of death caused by heart disease (n=34, 27.87%). In node 2, women who had an oxygen saturation of 93% and 95% had a mortality rate of 4.3%, node 3 shows that women who with an oxygen saturation of 95% and 96% had a death rate of 27.1%, and node 4 shows that women who had an oxygen saturation of >96% had the lowest mortality rates (12.9%; *χ*^2^_3_=140.7; *P*<.001).

Further, in the model, node 1 was split into 2 nodes, nodes 5 and 6, based on the pulse rate with a cutoff value of 97 beats per minute. Women who had a pulse rate of >97 beats per minute were at a higher risk of death (n=1257.14%; *χ*^2^_1_=10.8; *P*=.19). Node 2 split into 3 nodes based on age: nodes 7-9. Women who were older than 72 years were at a higher risk of death (n=45, 10.0%; *χ*^2^_3_=56.4; *P*<.001). Node 7 split into 2 nodes, nodes 12 and 13, based on SBP with a cutoff value of >187 mm Hg and a mortality rate of 8.5% (*χ*^2^_1_=23.4; *P*<.001). Node 4 split into 2 nodes, nodes 10 and 11, based on the pulse rate (heart rate) with a cutoff value of 97 beats per minute. Women with a pulse rate of >97 beats per minute had a higher mortality rate (n=8, 38.1%; *χ*^2^_1_=13.7; *P*=.004).

Node 9 split into 2 nodes based on medical diagnosis: nodes 14 and 15. Women diagnosed with congestive heart failure were at a higher risk of death (n=31,16.58%) than those with angina pectoris (n=14, 5.32%; *χ*^2^_1_=15.4; *P*<.001). Moreover, node 14 split into 2 nodes, nodes 16 and 17, based on pulse pressure with a cutoff value of 98 mm Hg. Women who had a pulse pressure of >98 mm Hg had a higher mortality rate (19.4%; *χ*^2^_1_=13.7; *P*=.002).

## Discussion

### Principal Findings

Several independent variables emerged as predictors of mortality caused by heart disease among the study participants, using the decision tree approach of the CHAID model. The CHAID model is a data mining technique that highly facilitates graphical presentation that provides an easy way for data interpretation in the medical field, with notable advantages over other models such as logistic regression analysis. In addition, it provides the ability to deal with multiple nodes, since it emerges all variables in a given data set [20].

Retrospective data were extracted from the EHRs and used to effectively predict death versus life status among Jordanian women who had heart disease. The built model predicts several predictor variables to identify women with heart disease who were at a high risk of death.

This study concluded that oxygen saturation (measured with pulse oximetry) is the most important predictor of death versus life status among female patients. We found that women with low tissue oxygen saturation had higher mortality rates than those with normal oxygen saturation. This finding is consistent with that of Cahyati et al [21] since they mentioned that lack of oxygenated blood in the myocardium is caused by atherosclerosis or plaque formation that blocks blood supply to the heart muscle. In turn, this leads to the formation of blood clots in the narrowing arteries, thus preventing blood flow to the cardiac system. This blockage can disrupt oxygen supply throughout the body, and the patient experiences shortness of breath, which, in turn, reduces oxygen saturation.

Second, pulse rate (heart rate) is the leading factor driving an increase in mortality among women. We found that women with a heart rate of >97 beats per minute are at a high risk of death. Many studies reported that heart rate is an independent risk factor for cardiovascular death [22,23]. Another large follow-up study and meta-analysis conducted by Tadic et al [24] supported the relationship between heart rate and cardiovascular morbidity and mortality among the general population; they reported that heart rate has a negative effect on both cardio- and cerebrovascular mortality, and that it is recommended to reduce the heart rate among these populations in order to prevent the primary and secondary effect of cardiac events.

The third factor was related to aging, which increases the risk of death, particularly among women. This result is consistent with those of other studies that explored the association between the aging process and the risk of death caused by cardiovascular disease. Rodgers et al [25] and Woodward [26] reported that age is an independent risk factor for the development of cardiovascular events owing to corresponding reductions in sex hormones (primarily estrogen), which plays an important role in protecting against heart disease.

Systolic blood pressure is the fourth leading cause of the increased risk of mortality among women. Our results show that any woman who has an SBP of >187 mm Hg was at a high risk of death due to heart disease. This finding paralleled that of Razo et al [27], who reported the substantial causal relationship between SBP and the development of ischemic heart disease at a cutoff point of 165 mm Hg. This finding could be attributed to the aging process that is associated with many devastating lifestyle changes such as increased sodium intake, decreased intake of fruits and vegetables, increase cholesterol level, and decreased physical activity, which lead to a substantial increase in SBP.

The fifth-ranking predictor, medical diagnosis of women, contributed as a risk factor of death since we found that women who had congestive heart failure were at higher risk of death than those who had coronary heart diseases such as angina pectoris and acute myocardial infarction. Many previous studies verified that female patients with heart failure experience persistent death and high mortality due to a reduced ejection fraction [28]. The statistical analysis conducted by the American Heart Association reported that heart attacks and coronary heart diseases were the main causes of death among individuals with cardiovascular disease [29].

As the sixth-ranking predictor of mortality risk among women, pulse pressure was the last factor in this study in the built model. Our model predicted that a pulse pressure of >98 mm Hg increased the risk of death among women. Previous reports have shown that pulse pressure is an important determinant of, and greatly influences, the development of heart diseases since it increases arterial stiffness resulting from the loss of elastin and collagen, leading to increases in the SBP and pulse pressure velocity [30,31].

### Implications in Clinical practice

Health care professionals must have a thorough understanding of the risk factors for heart disease in order to identify those women who are more susceptible to the development of heart disease and to implement the appropriate preventative measures. Outlined below are a few outcomes of risk factors in the treatment of heart disease.

#### Risk Assessment

Health care professionals can use risk assessment techniques to determine an individual woman's overall risk of developing heart disease. Oxygen saturation (measured with pulse oximetry), age, SBP, medical diagnoses, and pulse pressure are just a few of the risk factors taken into account by these instruments. By assessing a person's risk, medical professionals can determine whether additional diagnostic procedures and actions are necessary.

#### Patient Education

Finding and communicating risk factors for developing heart diseases in women is an essential part of clinical practice. Health care professionals can educate patients about risk factors that can be altered, such as blood pressure control and avoiding conditions that result in a particular medical diagnosis. They can provide guidance on how to implement necessary lifestyle changes such as adopting a heart-healthy diet, engaging in regular physical activity, quitting smoking, and skillfully managing stress.

#### Screening and Monitoring

Certain risk factors may necessitate routine screening and monitoring in clinical practice. This study provides critical proactive management strategies. For instance, patients with hypertension must routinely keep their blood pressure under control to prevent further complications of fatal heart diseases.

### Strengths

This study provides an important proposed model that can help physicians in precise decision-making that reflects the clinical consequences of the main risk factors for the development of cardiovascular events; thus, primary prevention strategies can be initiated to optimize the recurrence of cardiovascular events. Second, follow-up of the main variables and clinical features as predictors can be taken into account as a risk management strategy.

### Limitations

This study has the following limitations. First, missing, inconsistent, and noisy data were extracted from the EHS system. Second, several variables that could influence our results could not be obtained, such as smoking status, BMI, and the socioeconomic status of the population.

### Conclusions

Cardiovascular heart disease remains the leading cause of death globally. In this study, the most important variables were predicted using big data that were extracted from the clinical variables from the EHRs. The CHAID model as an MLA verified the accurate identification of the main predictors of cardiovascular mortality among women and can be used as a convenient tool for clinical prediction. Besides, follow-up of the main variables of oxygen saturation, pulse rate, SBP, and pulse pressure provides strategic measures of primary prevention of further complications of cardiovascular events.

## Appendix

Multimedia Appendix 1 Predictive model of death versus a life outcomes among Jordanian women.

## Abbreviations

AUC: area under the curve
CHAID: Chi-squared Automatic Interaction Detection
DBP: diastolic blood pressure
EHR: electronic health record
EHS: Electronic Health Solutions
FBS: fasting blood sugar
HbA_1c_: glycated hemoglobin
HDL: high-density lipoprotein
ICD-10: International Classification of Disease, Tenth Revision
LDH: lactate dehydrogenase
MAP: mean arterial pressure
MLA: machine learning algorithm
SBP: systolic blood pressure

## Data Availability

Data will be made available upon request while preserving the study participants’ privacy and confidentiality.
