# Supplementary figures and images for: Effective Prediction of Mortality by Heart Disease Among Women in Jordan Using the Chi-Squared Automatic Interaction Detection Model: Retrospective Validation Study
Source: JMIR Cardio. 2023 Jul 20;7:e48795. doi: 10.2196/48795 (PMC10401188; doi:10.2196/48795)

**Multimedia appendix 1.** Predictive model of death versus a life outcomes among Jordanian women.


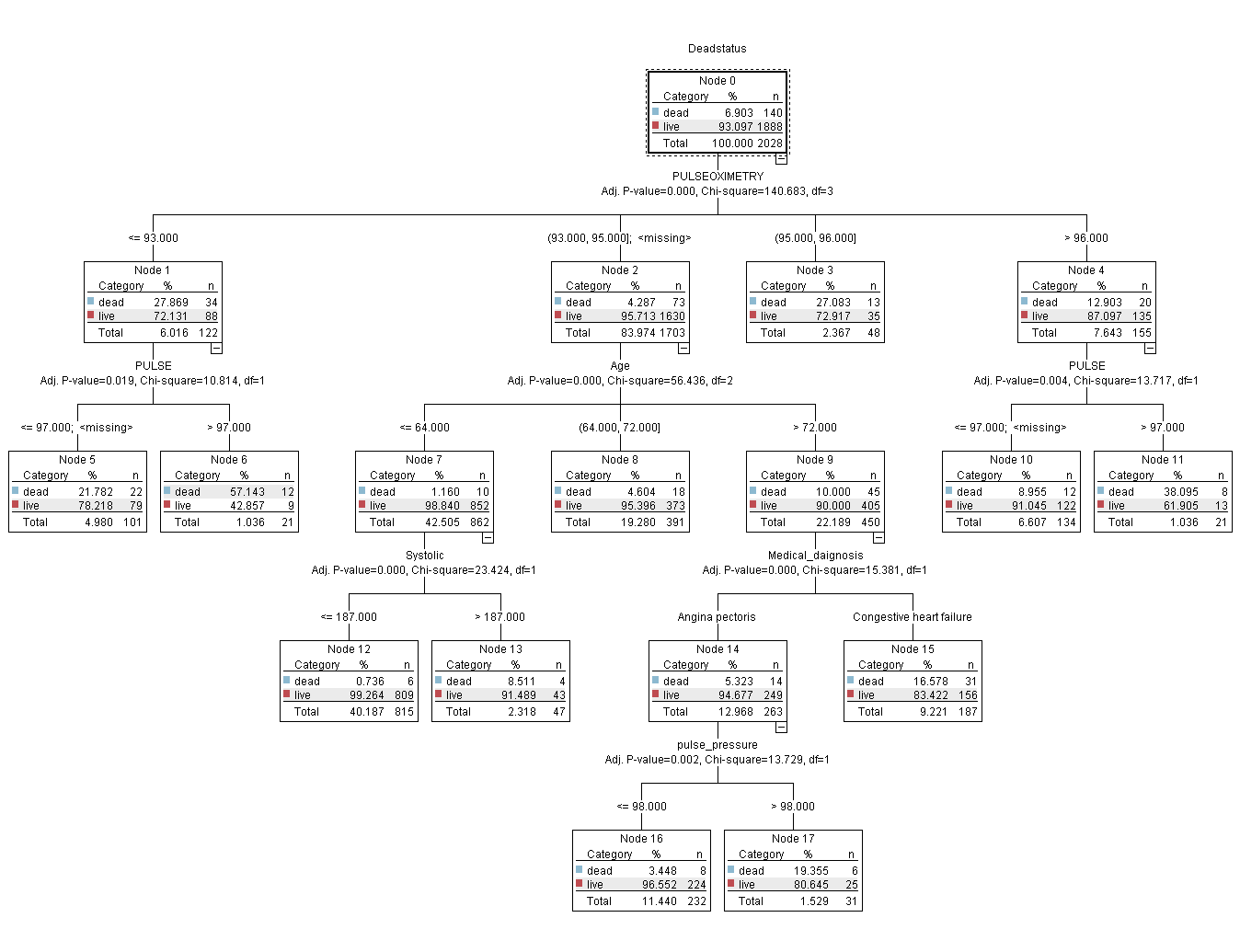

Supplement: Multimedia Appendix 1 [file cardio_v7i1e48795_app1.docx]
